# Phase 1 study of veliparib with carboplatin and weekly paclitaxel in Japanese patients with newly diagnosed ovarian cancer

**DOI:** 10.1111/cas.13381

**Published:** 2017-09-18

**Authors:** Shin Nishio, Munetaka Takekuma, Satoshi Takeuchi, Kouichirou Kawano, Naotake Tsuda, Kazuto Tasaki, Nobutaka Takahashi, Masakazu Abe, Aki Tanaka, Takayuki Nagasawa, Tadahiro Shoji, Hao Xiong, Silpa Nuthalapati, Terri Leahy, Hideyuki Hashiba, Tsukasa Kiriyama, Philip Komarnitsky, Yasuyuki Hirashima, Kimio Ushijima

**Affiliations:** ^1^ Department of Obstetrics and Gynecology Kurume University School of Medicine Kurume, Fukuoka Japan; ^2^ Division of Gynecology Shizuoka Cancer Center Nagaizumi, Shizuoka Japan; ^3^ Department of Obstetrics and Gynecology Iwate Medical University School of Medicine Morioka Iwate Japan; ^4^ AbbVie North Chicago Illinois USA; ^5^ AbbVie GK Tokyo Japan

**Keywords:** Japanese, ovarian cancer, poly(ADP‐ribose) polymerase inhibitors, phase I, veliparib

## Abstract

This phase 1, open‐label, dose‐escalation study was conducted to determine the safety, tolerability, pharmacokinetics and preliminary efficacy of veliparib with carboplatin and weekly paclitaxel in Japanese women with newly diagnosed, advanced ovarian cancer. Patients received veliparib at 100 or 150 mg b.i.d. on days 1–21 with carboplatin (area under the concentration–time curve 6 mg/mL•min) on day 1 and paclitaxel 80 mg/m^2^ on days 1, 8 and 15 every 3 weeks for up to 6 21‐day cycles. Dose escalation followed a 3 + 3 design to determine dose‐limiting toxicities, maximum tolerated dose and the recommended phase 2 dose. Nine patients (median age 62 [range 27–72] years) received a median of 5 (range 3–6) cycles of treatment (3 at 100 mg, 6 at 150 mg). There were no dose‐limiting toxicities. The most common adverse events of any grade were neutropenia (100%), alopecia (89%), peripheral sensory neuropathy (78%), and anemia, nausea and malaise (67% each). Grade 3 or 4 adverse events were associated with myelosuppression. Pharmacokinetics of carboplatin/paclitaxel were similar at both veliparib doses. Response, assessed in five patients, was partial in four and complete in one (objective response rate 100%). The response could not be assessed in four patients who had no measurable disease at baseline. The recommended phase 2 dose of veliparib, when combined with carboplatin/paclitaxel, is 150 mg b.i.d. Findings from this phase 1 trial demonstrate the tolerability and safety of veliparib with carboplatin/paclitaxel, a regimen with potential clinical benefit in Japanese women with ovarian cancer.

Each year, ovarian cancer is diagnosed in an estimated 225 000 women worldwide, most of whom (70%–85%) present with advanced disease.^1^ The current standard of care as first‐line treatment of advanced ovarian cancer is combination therapy with carboplatin and paclitaxel. However, development of recurrent or drug‐resistant disease in most women necessitates the use of alternative strategies, such as dose‐dense therapy, aimed at improving both progression‐free survival and overall survival.^2^, ^3^, ^4^, ^5^ To address the unmet need for novel strategies that deliver improved survival benefits, targeted agents are increasingly seen as an option for combination treatment with conventional cytotoxic chemotherapy.^6^

Veliparib (ABT‐888) is a potent, orally bioavailable poly(ADP‐ribose) polymerase (PARP)‐1/2 inhibitor that may increase the efficacy of chemotherapeutic regimens by delaying DNA repair after chemotherapy‐induced damage.^7^ In preclinical models, veliparib enhanced the activity of cisplatin, carboplatin and cyclophosphamide, whereas cell lines that quickly develop resistance to taxanes and carboplatin retained their sensitivity to veliparib, thus supporting the candidacy of this agent for the treatment of platinum‐resistant or taxane‐resistant ovarian cancer.^7^, ^8^ In phase 1 clinical trials, veliparib, either as a single‐agent therapy^9^ or in combination with cyclophosphamide^10^ or carboplatin and paclitaxel,^11^ had an acceptable tolerability profile. A phase 2 study conducted in patients with advanced or metastatic non‐small cell lung cancer similarly showed that veliparib in combination with carboplatin and paclitaxel was well tolerated and, compared with chemotherapy alone, showed a trend toward prolonging progression‐free and overall survival.^12^

Here we report the results of the first phase 1 study of continuously dosed veliparib administered with carboplatin and weekly paclitaxel in Japanese patients with ovarian cancer. The primary objective of this study was to confirm the recommended phase 2 dose (RP2D) of this regimen; secondary objectives were to assess the regimen's pharmacokinetics (PK) and safety, and the preliminary antitumor activity in this population.

## Materials and Methods

### Eligibility criteria

Women aged ≥20 years were eligible if they had newly diagnosed, chemotherapy‐naive, and histologically or cytologically confirmed (by International Federation of Gynecology and Obstetrics [FIGO] criteria) stage Ic–IV epithelial ovarian carcinoma, fallopian tube carcinoma, or primary peritoneal carcinoma and either optimal (<1 cm) residual disease or suboptimal residual disease. Patients were required to have undergone cytoreductive surgery within 1–12 weeks before study entry, to have an Eastern Cooperative Oncology Group (ECOG) performance status of 0 or 1, and to have adequate organ and marrow function, defined as absolute neutrophil count (ANC) ≥1500/mm^3^; platelet count ≥100 000/mm^3^; hemoglobin ≥9.5 g/dL; aspartate aminotransferase, alanine aminotransferase and alkaline phosphatase ≤2.5× the upper limit of normal (ULN); total bilirubin ≤1.5× ULN; albumin ≥3.0 g/dL; creatinine ≤1.0× ULN or creatinine clearance >60 mL/min (calculated by the Cockcroft–Gault formula); and peripheral neuropathy of grade <2. Patients who met any of the following criteria were excluded: history of invasive cancer within the past 3 years, prior radiotherapy or chemotherapy for any abdominal or pelvic tumor, any investigational agent within 4 weeks before enrollment or any Chinese anticancer medicine or herbal remedy within 14 days before enrollment, known sensitivity to any of the study medications, history or evidence of central nervous system disease, prior therapy with a PARP inhibitor, or any clinically significant uncontrolled condition.

### Study design and treatment schedule

This was a phase 1, open‐label, multicenter, dose‐escalation study to evaluate the tolerability, safety, PK and preliminary efficacy of oral veliparib in combination with carboplatin and weekly paclitaxel in Japanese patients with ovarian cancer. Patients were assigned to treatment with veliparib at either 100 mg (dose level 1 [DL1]) or 150 mg (DL2) b.i.d. on days 1–21 of a 21‐day cycle. In each dose group, veliparib was administered with intravenous carboplatin (area under the concentration–time curve [AUC] 6 mg/mL•min) on day 1 and with intravenous paclitaxel 80 mg/m^2^ on days 1, 8 and 15. Patients received up to 6 cycles of treatment unless they had disease progression or unmanageable toxicity. After completion of cycle 1, subsequent cycles were not begun until the patient's ANC was ≥1500/mm^3^ and the platelet count ≥100 000/mm^3^. Patients with ANC 1000–1499/mm^3^ or platelet count 75 000–99 000/mm^3^ (or both) could proceed to the next cycle of therapy with a predefined dose modification (Table S1). On day 8 or 15 of each cycle, another dose modification guideline was applied (Table S2). The patient's participation in the study was discontinued upon the third occurrence of hematologic toxicity that required dose modification per protocol. Treatment could be postponed for up to 21 days because of toxicity; longer toxicity‐related delay led to study discontinuation. Reduction of carboplatin and paclitaxel doses was permitted based on the toxicity observed (Table S3), which could necessitate discontinuation of either agent. At the investigator's discretion, patients who discontinued veliparib were required to discontinue the study but could continue to receive carboplatin and paclitaxel as standard of care. The trial was conducted in accordance with good clinical practice, the International Conference on Harmonization guidelines, and the 1964 Declaration of Helsinki and its later amendments; written informed consent was obtained from all patients before study enrollment.

### Dose escalation schedule

Dose escalation proceeded according to a 3 + 3 design to determine dose‐limiting toxicities (DLT), the maximum tolerated dose (MTD) and the RP2D. A minimum of 3 patients were enrolled at DL1. If none of these patients experienced a DLT, 3 more patients would be enrolled at DL2. If 1 or 2 patients experienced a DLT at DL1, 3 more patients would be enrolled at the same dose level. If ≤2 of 6 patients experienced a DLT, dose escalation would proceed to DL2. If ≥3 patients experienced a DLT, dose escalation would be stopped, with the following exceptions: (i) if 3 of 6 patients at DL1 experienced DLT, 3 more patients would enter the DL and the cohort be expanded to 9 patients, depending on a review of the specific DLT observed and discussion with the investigators; and (ii) if 3 of 9 patients at DL1 experienced DLT, escalation to DL2 would proceed, but if >3 of 9 patients experienced DLT, then dose escalation would stop.

### Dose‐limiting toxicity assessment and determination of RP2D

Dose‐limiting toxicities were determined based on events that occurred during the DLT evaluation period from day 1 of cycle 1 through pre‐dosing on day 1 of cycle 2. A patient was considered to be evaluable for DLT assessment if she had completed cycle 1 with the assigned regimen or had prematurely discontinued cycle 1 or experienced an adverse event (AE) related to veliparib. AE that occurred after the DLT evaluation period were considered in dose‐escalation decisions and tolerability assessment. The MTD was defined as the highest dose level at which ≤2 of 6 or ≤3 of 9 patients experienced a DLT. The RP2D was determined on the basis of assessment of the observed toxicities, the MTD, the overall safety profile of the regimen, and the PK of veliparib. In the event that the MTD was not reached, the RP2D was to be defined on the basis of safety and PK data and would not be higher than the maximum administered dose. Any of the following events that were considered to have a reasonable possibility of being related to the administration of veliparib were defined as DLT: grade 4 neutropenia lasting more than 7 days; grade 4 thrombocytopenia; febrile neutropenia; any grade ≥3 nonhematologic toxicities except nausea, vomiting and diarrhea; and transient metabolic toxicities such as glucose changes, hypokalemia or hypophosphatemia.

### Safety, pharmacokinetic and efficacy measurements

During the screening visit, a complete medical history (including a detailed oncology history) was collected. Study visits occurred on days 1, 8 and 15 of cycle 1 and each subsequent cycle; patients also attended for a final visit and a 30‐day follow‐up visit. AE monitoring was performed at each visit; most visits also included a physical examination and ECOG performance status evaluation. Toxicities were graded according to the NCI Common Terminology Criteria for Adverse Events version 4.03. For the determination of veliparib, carboplatin and paclitaxel PK, plasma samples were collected at multiple time points within 24 h of the first morning dose of veliparib on day 1 of cycle 1. The PK of carboplatin and paclitaxel were evaluated only with veliparib co‐administration. Values of the PK parameters of veliparib, carboplatin and paclitaxel, including the maximum observed plasma concentration (*C*_max_), time to *C*_max_ (*T*_max_) and AUC, were determined by using noncompartmental methods. In addition to tumor assessment at baseline, tumor response was assessed by using the Response Evaluation Criteria In Solid Tumors (RECIST) version 1.1^13^ every 9 weeks from day 1 of cycle 1 and at the final visit. The tumor marker cancer antigen 125 (CA‐125) was evaluated on day 1 of each cycle and at the final visit.

### Statistical analyses

The sample size was based on the toxicities observed during progression of the trial and on Japanese guidelines for the clinical evaluation of antitumor agents. No specific statistical hypotheses were planned, and descriptive statistics were used to analyze PK data. The safety population included all patients who received ≥1 dose of veliparib.

## Results

### Patient disposition and characteristics

Of 9 patients enrolled in the study who received ≥1 dose of veliparib, 3 entered DL1 (100 mg b.i.d.) and 6 entered DL2 (150 mg b.i.d.). Among 9 patients, 7 discontinued the study (2 of 3 at DL1 and 5 of 6 at DL2) because of AE (*n* = 6; DL1, 33%; DL2, 83%) or withdrawal of consent for reasons other than safety (*n* = 1; DL1, 33%). The patient who withdrew consent discontinued the study after receiving carboplatin and paclitaxel on day 1 of cycle 6 and, therefore, was tolerating treatment overall. The patient achieved complete response before withdrawal of consent, and refused further chemotherapy. No patient discontinued because of disease progression. Demographics and baseline characteristics of the safety population are summarized in Table 1. Briefly, patients' median age was 62 (range 27–72) years, most patients were younger than 65 years (56%) or had never smoked (78%), and the median duration since diagnosis of ovarian cancer was 0.95 (range 0.2–2.3) months. All patients had epithelial ovarian tumors, and at baseline most patients had an ECOG performance status of 0 (89%), measurable disease (56%) and a FIGO stage IIIc (56%).

**Table 1:** Patient demographics and baseline characteristics (safety population)

Characteristic	Veliparib		Total
	100 mg BID	150 mg BID	
	*n* = 3	*n* = 6	*N* = 9
Age, years			
Median (range)	65.0 (27–72)	59.5 (35–72)	62.0 (27–72)
<65, *n* (%)	1 (33)	4 (67)	5 (56)
≥65, *n* (%)	2 (67)	2 (33)	4 (44)
Race or ethnicity, *n* (%)			
Asian or Japanese	3 (100)	6 (100)	9 (100)
Median (range) disease duration, months	1.58 (1.3–2.3)	0.67 (0.2–2.0)	0.95 (0.2–2.3)
Smoking status, *n* (%)			
Former	0	2 (33)	2 (22)
Never	3 (100)	4 (67)	7 (78)
ECOG performance status, *n* (%)			
0	3 (100)	5 (83)	8 (89)
1	0	1 (17)	1 (11)
Measurable lesion at baseline, *n* (%)			
Any	2 (67)	3 (50)	5 (56)
None	1 (33)	3 (50)	4 (44)
FIGO stage at diagnosis, *n* (%)			
Ic	0	2 (33)	2 (22)
IIIa	0	1 (17)	1 (11)
IIIb	1 (33)	0	1 (11)
IIIc	2 (67)	3 (50)	5 (56)
Type of ovarian cancer, *n* (%)			
Epithelial ovarian	3 (100)	6 (100)	9 (100)
Histology, *n* (%)			
Serous	1 (33)	3 (50)	4 (44)
Endometrioid	1 (33)	1 (17)	2 (22)
Clear cell	1 (33)	1 (17)	2 (22)
Mixed	0	1 (17)	1 (11)

BID, twice daily; ECOG, Eastern Cooperative Oncology Group; FIGO, International Federation of Gynecology and Obstetrics.

### Exposure

Patients received a median of 5 (range 3–6) cycles of veliparib in combination with carboplatin and paclitaxel. No patient needed a veliparib dose reduction, but 7 of 9 (78%) needed ≥1 carboplatin dose reduction beginning at cycle 2 due to observed hematologic toxicities. Veliparib was delivered at a median dose intensity of 100% in all patients; carboplatin and paclitaxel were each delivered at a median dose intensity of ≥75%.

### Safety

All patients experienced ≥1 treatment‐emergent AE (TEAE) of any grade, and 7 of 9 (78%) experienced a grade 3 or 4 TEAE. The most common TEAE of any grade (regardless of relation to veliparib) were neutropenia (100%), alopecia (89%), peripheral sensory neuropathy (78%), and anemia, nausea and malaise (67% each). The most common grade 3 or 4 TEAE (regardless of relation to veliparib) included neutropenia (78%) and anemia (56%). Although hematologic toxicities were common, these toxicities were manageable with medication, dose reductions or dose delays. One patient at DL2 (17%) experienced a treatment‐emergent serious AE (vomiting). The patient received antiemetic therapy with metoclopramide (5 and 10 mg as needed) and domperidone (60 mg as needed). Additional treatment details are reported in Table S4. This event was considered by the investigators to have at least a reasonable possibility of being related to any of the study treatments, including veliparib. Veliparib was discontinued because of a TEAE by 6 of 9 (67%) patients overall. TEAE leading to veliparib discontinuation were neutropenia, in 1 of 3 (33%) patients at DL1 and in 5 of 6 (83%) patients at DL2, and thrombocytopenia, in 1 of 6 (17%) patients at DL2. Time of onset, grade and duration of TEAE leading to discontinuation are reported in Table S5. Patients who discontinued veliparib due to a third occurrence of hematologic toxicity came off the study. However, at the investigator's discretion, all could continue to receive carboplatin and paclitaxel as standard of care because hematologic toxicity was not so critical. No DLT were observed at any dose level. There were no AE that led to death. All TEAE that occurred in ≥20% of patients are summarized in Table 2. The MTD of the regimen was not reached. Therefore, the RP2D of veliparib 150 mg b.i.d. in combination with carboplatin and weekly paclitaxel was determined on the basis of the safety and PK data.

**Table 2:** Treatment‐emergent adverse events (TEAE) occurring in ≥20% of patients (safety population)

TEAE ≥20%, *n* (%)	Veliparib				Total	
	100 mg BID		150 mg BID			
	*n* = 3		*n* = 6		*N* = 9	
	All	Grade 3 or 4	All	Grade 3 or 4	All	Grade 3 or 4
Any AE	3 (100)	3 (100)	6 (100)	4 (67)	9 (100)	7 (78)
Neutropenia†	3 (100)	3 (100)	6 (100)	4 (67)	9 (100)	7 (78)
Alopecia	3 (100)	0	5 (83)	0	8 (89)	0
Peripheral sensory neuropathy‡	2 (67)	0	5 (83)	0	7 (78)	0
Anemia	2 (67)	1 (33)	4 (67)	4 (67)	6 (67)	5 (56)
Nausea	2 (67)	0	4 (67)	0	6 (67)	0
Malaise	3 (100)	0	3 (50)	0	6 (67)	0
Thrombocytopenia§	1 (33)	0	4 (67)	1 (17)	5 (56)	1 (17)
Constipation	2 (67)	0	3 (50)	0	5 (56)	0
Vomiting	1 (33)	0	3 (50)	0	4 (44)	0
Dysgeusia	1 (33)	0	3 (50)	0	4 (44)	0
Leukopenia¶	2 (67)	2 (67)	2 (33)	2 (33)	4 (44)	4 (44)
Decreased appetite	2 (67)	0	1 (17)	0	3 (33)	0
Stomatitis	0	0	2 (33)	0	2 (22)	0
Nasopharyngitis	1 (33)	0	1 (17)	0	1 (22)	0
Arthralgia	1 (33)	0	1 (17)	0	2 (22)	0
Dry skin	0	0	2 (33)	0	2 (22)	0
Nail disorder	0	0	2 (33)	0	2 (22)	0
Rash	0	0	2 (33)	0	2 (22)	0

†Including neutrophil count decreased. ‡Including neuropathy peripheral. §Including platelet count decreased. ¶Including white blood cell count decreased. BID, twice daily; TEAE, treatment‐emergent adverse event.

### Pharmacokinetics

**Figure 1:**
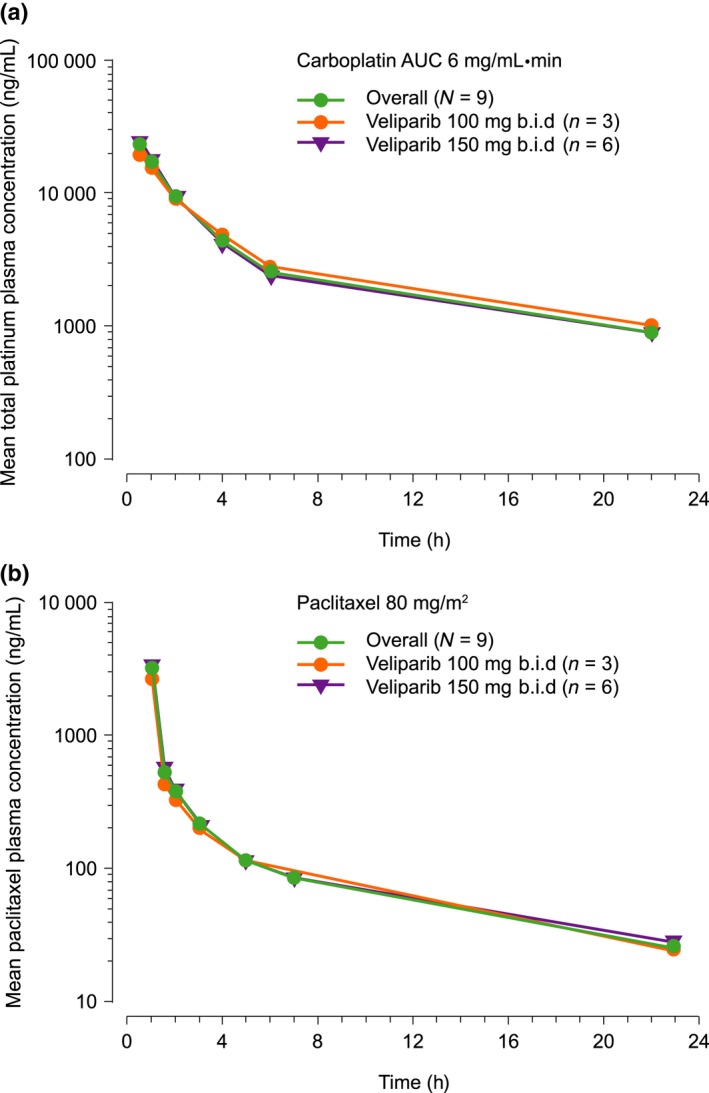
Mean plasma concentration–time profiles of (a) carboplatin and (b) paclitaxel. AUC, area under the plasma concentration–time curve.

The dose‐normalized *C*_max_ and AUC values for veliparib were comparable in the two veliparib dose cohorts (Table 3). The plasma concentration–time profiles of carboplatin and paclitaxel were similar in the two veliparib dose cohorts (Fig. 1). The mean (SD) *C*_max_ and AUC from time zero to infinity (AUC_∞_) for carboplatin, measured as total platinum, were 23.3 (4.28) μg/mL and 5.30 (0.491) mg•min/mL, respectively. The mean (SD) *C*_max_ and AUC_∞_ for paclitaxel were 3.30 (0.644) μg/mL and 5.32 (0.952) μg•h/mL, respectively. The PK of carboplatin and paclitaxel were similar at the two veliparib dose levels.

**Table 3:** Pharmacokinetic parameters of veliparib on day 1 of cycle 1

Veliparib dose, mg	*N*	*T*_max_, h	*C*_max_/dose, ng/mL/mg	AUC_0–8_/dose, ng•h/mL/mg
100	3	4.0 (1.0–4.0)	9.73 ± 1.63	37.2 ± 4.37
150	6	3.0 (1.0–4.4)	7.19 ± 1.16	36.7 ± 8.10

All other parameters are presented as mean ± SD. AUC_0–8_, area under the concentration–time curve from time 0 to 8 h; *C*_max_, maximum plasma concentration; *T*_max_, time to *C*_max_. *T*_max_ is presented as median (range).

### Efficacy

**Figure 2:**
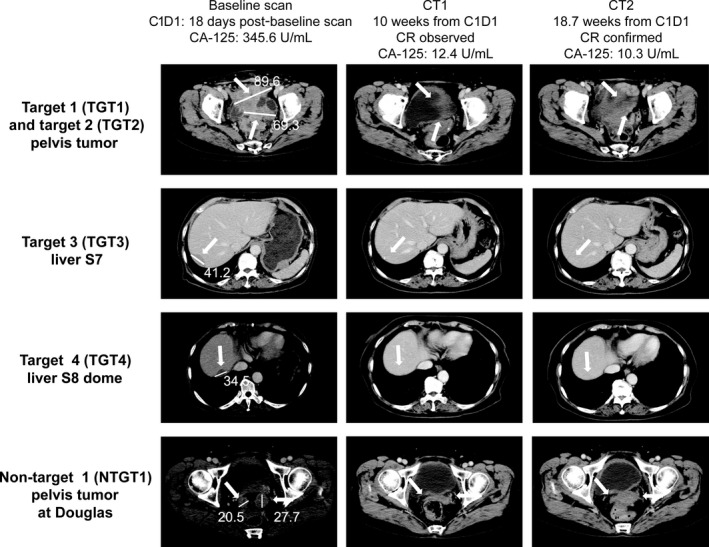
Computed tomography images showing a complete response to veliparib 100 mg b.i.d. plus carboplatin and paclitaxel in a single patient. C1D1, cycle 1, day 1; CA‐125, cancer antigen 125; CR, complete response; CT, computed tomography.

**Figure 3:**
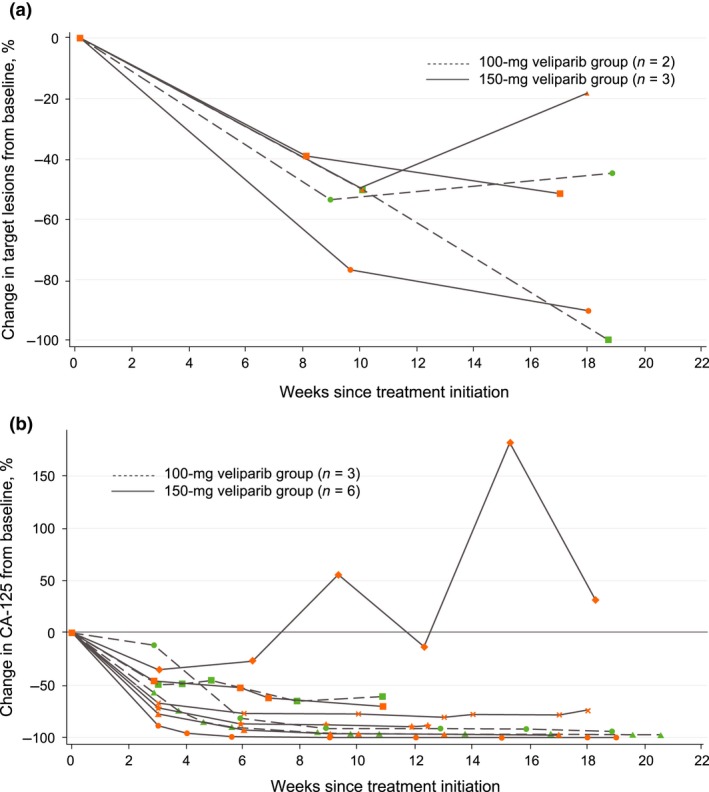
Median best percentage change from baseline in (a) the size of target lesions and (b) CA‐125.

In 5 patients in whom the response to treatment with veliparib plus carboplatin and paclitaxel was assessed, the objective response rate was 100%. Response could not be assessed in 4 patients because they had no measurable disease at baseline as a result of complete resection of the tumor during primary cytoreductive surgery. One patient had a complete response (veliparib 100 mg b.i.d. cohort) and 4 patients had a partial response (1 to veliparib 100 mg b.i.d. and 3 to veliparib 150 mg b.i.d.). Computed tomography images of the patient with a complete response are shown in Figure 2. The median best percentage change from baseline over time in the sum of target lesions was −76.75% (range −100.00% to −53.51%) in the veliparib 100 mg b.i.d. cohort and −51.57% (range −90.29% to −50.00%) in the veliparib 150 mg b.i.d. cohort. The overall median best percentage change was −53.51% (range −100.00% to −50.00%) (Fig. 3a). A CA‐125 response according to the Gynecologic Cancer InterGroup definition was achieved in 8 of 9 (89%) patients. The median best percentage change from baseline over time in CA‐125 expression was −94.24% (range −97.31% to −65.25%) in the veliparib 100 mg b.i.d. cohort, −84.87% (range −99.79% to −34.96%) in the veliparib 150 mg b.i.d. cohort, and −89.33% (range −99.79% to −34.96%) overall (Fig. 3b).

## Discussion

The inhibition of PARP is a promising therapeutic strategy for the treatment of cancers with specific DNA‐repair defects: it offers robust antitumor activity and has few of the tolerability issues associated with conventional chemotherapy.^14^ PARP inhibitors are particularly active in cells with an impaired homologous‐recombination repair pathway, as commonly found in a significant proportion of *BRCA*‐wildtype and *BRCA*‐mutated ovarian cancers.^15^, ^16^, ^17^, ^18^ PARP inhibition has yielded durable responses in patients with platinum‐sensitive and platinum‐resistant ovarian cancer; the frequency of such responses correlates with the platinum‐free interval.^19^ PARP inhibitors have shown clinical benefit in patients with platinum‐sensitive, recurrent ovarian cancer,^17^, ^18^ and the PARP inhibitor olaparib has been approved by the European Medicines Agency and the FDA for the treatment of *BRCA*‐deficient ovarian cancer.^20^, ^21^ Rucaparib and niraparib are also FDA approved.^22^, ^23^ Recent promising data have increased attention on combination strategies that include PARP inhibitors.

The findings of this phase 1 study demonstrate that in Japanese women with advanced ovarian cancer, the combination of veliparib with carboplatin and paclitaxel had a manageable safety profile at a veliparib dose of 100 or 150 mg b.i.d. Rates of early discontinuation due to hematologic toxicity were consistent with previous observations in a study conducted in Japanese patients with advanced ovarian cancer treated with first‐line carboplatin and paclitaxel.^2^ Dose reductions of carboplatin were implemented because of hematologic toxicity. Results from two placebo‐controlled phase 2 studies indicate that hematologic toxicity of veliparib plus carboplatin/paclitaxel was comparable to that of placebo plus carboplatin/paclitaxel (excluding leukopenia).^12^, ^24^ Therefore, when such toxicity was observed in the reported trial, the dose of carboplatin was reduced, because prior results supported that hematologic toxicity was mainly due to carboplatin, not veliparib. No DLT were observed at either dose level. As the MTD of the regimen was not reached, the RP2D of veliparib 150 mg b.i.d. in combination with carboplatin and weekly paclitaxel was determined on the basis of the safety and PK data. The most common treatment‐related toxicities were alopecia, anemia, neutropenia, nausea, malaise and peripheral sensory neuropathy. Grade 3 and 4 AE were predominantly associated with myelosuppression. Other studies have shown that myelosuppression appears to be increased when PARP inhibitors are added to chemotherapy, although the extent of increased myelosuppression may be related to the schedule of administration, typically limited to a 5‐day or 7‐day course every 3 or 4 weeks.^25^, ^26^, ^27^ In the present study, continuous twice‐daily administration of veliparib did not cause unmanageable hematologic toxicity. One patient experienced a treatment‐emergent SAE of vomiting that was grade 1, but was considered an SAE because the patient required prolonged hospitalization. As supportive therapy, the patient was treated with metoclopramide, B fluid (rehydration solution including amino acids with electrolytes/glucose/Vitamin B1) and domperidone. No action was taken with the study drug regarding the event of vomiting, which resolved on day 3 (cycle 1, day 3); the patient was discharged on day 4 (cycle 1, day 4).

Exposures of carboplatin and dose‐normalized veliparib were comparable to those observed in a similar study in Japanese patients with non‐small cell lung cancer who received veliparib 40–120 mg.^11^ Similarly, the *C*_max_ and AUC values for paclitaxel at the administered dose of 80 mg/m^2^ were consistent with those reported in the literature.^28^, ^29^ A cross‐study comparison suggests that there is no PK interaction between veliparib and carboplatin or paclitaxel.

Efficacy findings were encouraging: response (per RECIST version 1.1^13^) occurred in all patients with measurable disease at baseline and was complete in 1 patient and partial in 4; all but 1 patient had a CA‐125 response by the Gynecologic Cancer InterGroup definition. Although high response rates are expected in previously untreated and unselected patients with advanced epithelial ovarian tumors treated with paclitaxel/platinum induction therapy, the 100% objective response rate in this study is encouraging and exceeds the 68%–75% response rates associated with paclitaxel/platinum reported from other studies.^30^, ^31^, ^32^ In another phase 1 study in Japanese patients with non‐small cell lung cancer and no more than 1 prior line of treatment, the combination of veliparib with carboplatin/paclitaxel also showed encouraging antitumor activity, with an objective response rate of 55%.^11^

Any conclusions of this study are limited by the small sample size, the nonrandomized design and absence of a control group, and the lack of any formal hypothesis testing. Nevertheless, the safety and efficacy findings are encouraging and warrant continued investigation of this regimen. A multinational phase 3 study (NCT02470585 at ClinicalTrials.gov) is investigating veliparib as both induction (with carboplatin/paclitaxel) and continuation maintenance therapy (as a single agent) in women with newly diagnosed stage III or IV epithelial ovarian, fallopian tube or primary peritoneal cancer. The data from this phase 1 trial of veliparib combined with carboplatin/paclitaxel demonstrate that this regimen is well tolerated and has potential for clinical benefit in Japanese women with ovarian cancer.

## Disclosure Statement

HX, SN, TL and PK were employed by AbbVie; HX, SN, TL and PK are authors who own AbbVie stock. HH is employed by AbbVie GK (Japan). TK is employed by AbbVie. YH received grants from AstraZeneca, AbbVie GK, Eisai, Ono Pharmaceutical, Taiho Pharmaceutical and Takeda Pharmaceutical. KU received honoraria from Chugai Pharmaceutical, and research funding from Takeda Pharmaceutical, Ono Pharmaceutical, AbbVie GK, Eisai, MSD K.K. and Pfizer. The remaining authors have nothing to disclose.

## Supporting information


**Table S1.** Dose modification for dose‐restricting hematologic toxicity, reduced ANC (1000–1499/mm^3^), or reduced platelets (75 000–99 000/mm^3^) on day 1 of each cycle.

**Table S2.** Dose modifications for hematologic toxicity on day 8 or 15 of each cycle.

**Table S3.** Criteria for treatment interruption based on delayed hematologic recovery and nonhematologic toxicity.

**Table S4.** Medications and supplements for patient with serious adverse event (SAE) of vomiting.

**Table S5.** Time of onset, grade, and duration of treatment‐emergent adverse events (TEAE) for patients withdrawn from study.
