# Supplementary material for: Phase 1 study of veliparib with carboplatin and weekly paclitaxel in Japanese patients with newly diagnosed ovarian cancer
Source: Cancer Sci. 2017 Sep 18;108(11):2213–20. doi: 10.1111/cas.13381 (PMC5665762; doi:10.1111/cas.13381)
Supplement: Supplementary file 1 — Table S1. Dose modification for dose‐restricting hematologic toxicity, reduced ANC (1000–1499/mm3), or reduced platelets (75 000–99 000/mm3) on day 1 of each cycle. [file CAS-108-2213-s001.pdf]

Supporting Information:

Supplemental Table

Table S1. Dose modification for dose-restricting hematologic toxicity, reduced ANC (1,000–1,499/mm^3), or reduced platelets (75,000–99,000/mm^3) on day 1 of each cycle

ANC	PLT	First occurrence	Second occurrence	Third occurrence
Yes	No	Reduce carboplatin 1 AUC unit (AUC 5) and add G-CSF	Discontinue day 15 paclitaxel dose	Discontinue veliparib and notify AbbVie Medical Monitor
Yes	Yes	Reduce carboplatin 1 AUC unit (AUC 5) and add G-CSF	Reduce carboplatin 1 AUC unit (AUC 4) and discontinue day 15 paclitaxel dose	Discontinue veliparib and notify AbbVie Medical Monitor
No	Yes	Reduce carboplatin 1 AUC unit (AUC 5)	Reduce carboplatin 1 AUC unit (AUC 4)	Discontinue veliparib and notify AbbVie Medical Monitor

For patients who had 2 dose reductions for ANC only and then developed thrombocytopenia only, an additional dose modification was allowed, after discussion with AbbVie Medical Monitor.
ANC, absolute neutrophil count; AUC, area under the concentration-time curve; G-CSF, granulocyte colony-stimulating factor.
