# Supplementary material for: Phase 1 study of veliparib with carboplatin and weekly paclitaxel in Japanese patients with newly diagnosed ovarian cancer
Source: Cancer Sci. 2017 Sep 18;108(11):2213–20. doi: 10.1111/cas.13381 (PMC5665762; doi:10.1111/cas.13381)
Supplement: Supplementary file 2 — Table S2. Dose modifications for hematologic toxicity on day 8 or 15 of each cycle. [file CAS-108-2213-s002.pdf]

Supporting Information:

Supplemental Table

Table S2. Dose modifications for hematologic toxicity on day 8 or 15 of each cycle

**ANC < 500**	**PLT < 50,000**	First occurrence	Second occurrence	Third occurrence
Yes	No	Reduce carboplatin one AUC unit (AUC 5) and add G-CSF with the next cycle	Discontinue Day 15 paclitaxel dose with the next cycle	Discontinue veliparib and notify AbbVie Medical Monitor
Yes	Yes	Reduce carboplatin one AUC unit (AUC 5) and add G-CSF with the next cycle	Reduce carboplatin one AUC unit (AUC 4) and discontinue Day 15 paclitaxel dose with the next cycle	Discontinue veliparib and notify AbbVie Medical Monitor
No	Yes	Reduce carboplatin one AUC unit (AUC 5) with the next cycle	Reduce carboplatin one AUC unit (AUC 4) with the next cycle	Discontinue veliparib and notify AbbVie Medical Monitor

For patients who have had 2 dose reductions for ANC only and then develop thrombocytopenia only, an additional dose modification is allowed, but should be discussed with AbbVie Medical Monitor.
ANC, absolute neutrophil count; AUC, area under the concentration-time curve; G-CSF, granulocyte colony-stimulating factor.
