# Supplementary material for: Phase 1 study of veliparib with carboplatin and weekly paclitaxel in Japanese patients with newly diagnosed ovarian cancer
Source: Cancer Sci. 2017 Sep 18;108(11):2213–20. doi: 10.1111/cas.13381 (PMC5665762; doi:10.1111/cas.13381)
Supplement: Supplementary file 3 — Table S3. Criteria for treatment interruption based on delayed hematologic recovery and nonhematologic toxicity. [file CAS-108-2213-s003.pdf]

Supporting Information:

Supplemental Table

Table S3. Criteria for treatment interruption based on delayed hematologic recovery and nonhematologic toxicity

Category	Criteria
Neutropenia	ANC <1,000 cells/mm^3 within 24 hours prior to day 1 of each cycle of scheduled therapy
Thrombocytopenia	Platelet count <75,000/mm^3 within 24 hours prior to day 1 of each cycle of scheduled therapy
Peripheral neuropathy grade ≥2	Reduction of 1 dose level in paclitaxel and delay in all subsequent protocol-directed therapy for a maximum of 3 weeks until recovery to grade 1
Hepatic toxicity	Development of grade ≥3 elevations in SGOT (AST), SGPT (ALT), alkaline phosphatase, or bilirubin require reduction of 1 dose level in paclitaxel and delay in subsequent therapy for a maximum of 3 weeks until recovery to grade ≤1
ALT, alanine aminotransaminase; ANC, absolute neutrophil count; AST, aspartate aminotransferase; SGOT, serum glutamic oxaloacetic transaminase; SGPT, serum glutamic pyruvic transaminase.	
