# Supplementary material for: Phase 1 study of veliparib with carboplatin and weekly paclitaxel in Japanese patients with newly diagnosed ovarian cancer
Source: Cancer Sci. 2017 Sep 18;108(11):2213–20. doi: 10.1111/cas.13381 (PMC5665762; doi:10.1111/cas.13381)
Supplement: Supplementary file 4 — Table S4. Medications and supplements for patient with serious adverse event (SAE) of vomiting. [file CAS-108-2213-s004.pdf]

Supporting Information:

Supplemental Table

Table S4. Medications and supplements for patient with SAE of vomiting

EPOCH			Rx day		Medication (Generic Name)	D: Dose (unit)<br>F: Frequency<br>R: Route	Reason for use
Name	Interval	Day	Start	End			
Pre	To -1		-3	Ongoing	Ketoprofen	D: 40 mg<br>F: PRN<br>R: Topical	Lumbago
Cycle1	1 to 21	1	1	56	Magnesium Oxide	D: 330 mg<br>F: TID<br>R: Oral	Constipation
			1	141	Palonosetron	D: 0.75 mg<br>F: Other – every chemotherapy<br>R: Intravenous drip	Pre-infusion medication
			1	141	Dexamethasone	D: 19.8 – 6.6 mg<br>F: Other – every chemotherapy<br>R: Intravenous drip	Pre-infusion medication
			1	141	Dexchlorpheniramine	D: 5 mg<br>F: Other – every chemotherapy<br>R: Intravenous drip	Pre-infusion medication
			1	141	Ranitidine	D: 50 mg<br>F: Other – every chemotherapy<br>R: Intravenous drip	Pre-infusion medication
		2	2	3	Diastase	D: 1.3 mg<br>F: PRN<br>R: Oral	Nausea
			2	56	Metoclopramide	D: 5 mg<br>F: PRN<br>R: Oral	Nausea

		3	3	3	B Fluid (amino acids with electrolytes/glucose/Vitamin B1)	D: 500 ml<br>F: BID<br>R: Intravenous drip	Prevention of dehydration
			3	3	Domperidone	D: 60 mg<br>F: PRN<br>R: Rectal	Nausea, vomiting
			3	3	Metoclopramide	D: 10 mg<br>F: PRN<br>R: Intravenous bolus	Nausea, vomiting
		4	4	10	Dexamethasone	D: 2.0 mg<br>F: BID<br>R: Oral	Nausea
2	22 to 42	2	23	25	Dexamethasone	D: 2.0 mg<br>F: BID<br>R: Oral	Nausea
		9	30	32	Dexamethasone	D: 2.0 mg<br>F: PRN<br>R: Oral	Nausea
3	43 to 70	2	44	46	Dexamethasone	D: 2.0 mg<br>F: PRN<br>R: Oral	Nausea
		17	59	61	Dexamethasone	D: 2.0 mg<br>F: PRN<br>R: Oral	Prevention of nausea
			59	61	Filgrastim	D: 75 mcg<br>F: QD<br>R: Subcutaneous	Neutropenia on C3D1

C, cycle; BID, twice a day; D, day; PRN, as needed; QD, once a day; Rx, prescription; SAE, serious adverse event; TID, three times a day.
