# Supplementary material for: Phase 1 study of veliparib with carboplatin and weekly paclitaxel in Japanese patients with newly diagnosed ovarian cancer
Source: Cancer Sci. 2017 Sep 18;108(11):2213–20. doi: 10.1111/cas.13381 (PMC5665762; doi:10.1111/cas.13381)
Supplement: Supplementary file 5 — Table S5. Time of onset, grade, and duration of treatment‐emergent adverse events (TEAE) for patients withdrawn from study. [file CAS-108-2213-s005.pdf]

Supporting Information:

Supplemental Table

Table S5. Time of onset, grade, and duration of TEAEs for patients withdrawn from study

<table>
<tr><th rowspan="4">Dose level</th><th colspan="6">Occurrence of hematologic toxicity requiring dose modification</th><th rowspan="2">Neutropenia</th><th rowspan="2">Thrombocytopenia</th></tr>
<tr><th colspan="2">First</th><th colspan="2">Second</th><th colspan="2">Third</th></tr>
<tr><th>Neutrophil (grade)</th><th>Platelet (grade)</th><th>Neutrophil (grade)</th><th>Platelet (grade)</th><th>Neutrophil (grade)</th><th>Platelet (grade)</th><th rowspan="2">Worst grade (duration of grade ≥3)</th><th rowspan="2">Worst grade (duration of grade ≥2)</th></tr>
<tr><th colspan="2">Time of onset</th><th colspan="2">Time of onset</th><th colspan="2">Time of onset</th></tr>
<tr><td rowspan="2">1</td><td>2</td><td>0</td><td>2</td><td>1</td><td>2</td><td>1</td><td rowspan="2">3
(20 days, 7 days)</td><td rowspan="2">1
(none)</td></tr>
<tr><td colspan="2">C2D1</td><td colspan="2">C3D1</td><td colspan="2">C4D1</td></tr>
<tr><td rowspan="2">2</td><td>3</td><td>1</td><td>0</td><td>1</td><td>2</td><td>1</td><td rowspan="2">3
(11 days, 6 days, 7 days)</td><td rowspan="2">2
(7 days)</td></tr>
<tr><td colspan="2">C2D1</td><td colspan="2">C4D1</td><td colspan="2">C2D1</td></tr>
<tr><td rowspan="2">2</td><td>2</td><td>1</td><td>4</td><td>0</td><td>3</td><td>0</td><td rowspan="2">4
(42 days)</td><td rowspan="2">3
(7 days)</td></tr>
<tr><td colspan="2">C2D1</td><td colspan="2">C2D8</td><td colspan="2">C4D1</td></tr>
<tr><td rowspan="2">2</td><td>2</td><td>0</td><td>0</td><td>1</td><td>2</td><td>1</td><td rowspan="2">3
(7 days)</td><td rowspan="2">2
(7 days, 7 days)</td></tr>
<tr><td colspan="2">C4D1</td><td colspan="2">C5D1</td><td colspan="2">C6D1</td></tr>
<tr><td rowspan="2">2</td><td>2</td><td>0</td><td>2</td><td>1</td><td>2</td><td>1</td><td rowspan="2">3
(7 days, 7 days)</td><td rowspan="2">1
(none)</td></tr>
<tr><td colspan="2">C2D1</td><td colspan="2">C4D1</td><td colspan="2">C5D1</td></tr>
<tr><td rowspan="2">2</td><td>2</td><td>1</td><td>2</td><td>1</td><td>2</td><td>1</td><td rowspan="2">2
(none)</td><td rowspan="2">2
(7 days, 7 days, 7 days)</td></tr>
<tr><td colspan="2">C3D1</td><td colspan="2">C4D1</td><td colspan="2">C6D1</td></tr>
<tr><td colspan="9">Grey cells indicate which hematologic toxicity (neutropenia, thrombocytopenia, or both) requires protocol-defined dose modification.
C, cycle; D, day; TEAE, treatment-emergent adverse event.</td></tr>
</table>
